# Association between white matter hyperintensities and gray matter volume in cerebral small vessel disease: insights from periventricular and deep white matter lesions

**DOI:** 10.3389/fneur.2025.1590997

**Published:** 2025-07-09

**Authors:** Weishun Feng, Xinjun Lei, Shida Xu, Zhihua Xu

**Affiliations:** ^1^Department of Radiology, Lishui Hospital of Traditional Chinese Medicine Affiliated to Zhejiang Chinese Medical University, Lishui, China; ^2^Department of Radiology, Tongde Hospital of Zhejiang Province, Hangzhou, China

**Keywords:** white matter hyperintensity, periventricular white matter, cerebral small vessel disease, gray matter, atrophy

## Abstract

**Objective:**

To investigate the relationship between the severity of periventricular white matter hyperintensities (PWMH) and deep white matter hyperintensities (DWMH) and gray matter volume in cerebral small vessel disease (CSVD) patients.

**Methods:**

Clinical and imaging data from 125 CSVD patients, collected between April and December 2022, were analyzed. The severity of PWMH and DWMH was assessed using the Fazekas scale. 3D T1-weighted images were processed with FSL software to segment gray matter and intracranial volume. The normalized gray matter volume (GM_n) was computed as the proportional ratio of gray matter volume relative to total intracranial volume.

**Results:**

Group-level analysis revealed significant differences in GM_n were observed across various Fazekas scores for both DWMH (*p* = 0.041) and PWMH (*p* < 0.001). *Post hoc* analysis with false discovery rate (FDR) correction showed that PWMH severity was associated with significantly lower GM_n in scores 2 and 3 compared to score 1 (FDR-adjusted *p* = 0.001 and 0.004, respectively), though no difference was observed between scores 2 and 3 (*p* > 0.05). For DWMH, no pairwise comparisons survived FDR correction, though a non-significant trend toward reduced GM_n was noted in score 3 versus scores 0–1 (FDR-adjusted *p* = 0.08). After multivariate analysis adjusting for vascular risk factors, PWMH severity was found to be independently associated with GM_n [*p* < 0.05, *β* (95% CI): Age −0.212 (−0.412, −0.011), PWMH score −0.408 (−0.605, −0.210)], while DWMH severity showed no independent association with GM_n (*p* > 0.05). Mediation analysis revealed PWMH significantly mediated 39.1% of the total effect of age on GM_n (indirect effect = −0.136; 95% CI: −0.265 to −0.053).

**Conclusion:**

PWMH, but not DWMH, is independently associated with gray matter volume and partially mediates the age-related decline in GM volume. These findings highlight the topographical and mechanistic heterogeneity of WMH subtypes and underscore PWMH as a potential imaging marker for early intervention in CSVD-related neurodegeneration.

## Introduction

White matter hyperintensities (WMH), one of the typical imaging biomarkers of cerebral small vessel disease (CSVD) (1), are highly prevalent in aging population and serve as critical risk factors for stroke, cognitive decline, and dementia (2–4). Simultaneously, gray matter (GM) atrophy—another hallmark of aging and neurodegeneration—has been widely observed in conditions such as Alzheimer’s disease (5, 6) and Parkinson’s disease (7, 8). Mounting evidence suggests that vascular pathology and neurodegeneration are interrelated processes, and that structural damage in white matter may contribute to GM atrophy (9). Understanding this relationship holds clinical significance for early diagnosis, disease monitoring, and the development of targeted interventions for aging-related cognitive disorders.

The biological mechanisms linking WMH and GM atrophy are increasingly recognized but remain incompletely understood. Several hypotheses have been proposed, including the disruption of the connecting white matter tracts (10, 11), microvascular hypoperfusion (11–13), metabolic insufficiency (11, 12), iron burden (14) and APOE4 carrier status (15). These processes may ultimately lead to reduced GM synaptic input, neuroinflammation, metabolic insufficiency, and neuronal loss. However, the extent and pattern of gray matter damage may vary depending on the spatial distribution of WMH.

WMHs are anatomically heterogeneous and are often subclassified into periventricular WMH (PWMH) and deep WMH (DWMH) based on their location relative to the lateral ventricles (1). These subtypes may reflect distinct vascular territories, underlying pathologies, and functional implications (16). PWMHs typically lie along the ventricular surface and overlap with long-range projection fibers, while DWMHs affect more localized white matter tracts. Despite this anatomical distinction, most prior imaging studies have assessed the relationship between WMH and gray matter (GM) atrophy using total WMH burden to examine associations with GM atrophy (17, 18), thereby potentially overlooking subtype-specific effects. Emerging clinical evidence has indicated that PWMH—but not DWMH—is independently associated with cognitive impairment (19, 20). This discrepancy raises the possibility that PWMH and DWMH differ not only in location but also in their functional consequences and impact on GM structure. However, direct comparisons of their respective effects on GM volume remain limited. Additionally, the potential mediating role of WMH in age-related gray matter decline—particularly when stratified by WMH subtype—has rarely been explored.

To address these gaps, the present study aimed to investigate the associations between PWMH and DWMH severity (using the Fazekas scale) and normalized gray matter volume (GM_n) in CSVD patients. We further sought to evaluate whether PWMH acts as a mediator in the relationship between age and GM atrophy. By distinguishing the roles of WMH subtypes, this study seeks to clarify the structural pathways through which vascular burden contributes to neurodegeneration, and to provide insight into more precise imaging biomarkers for aging-related brain disease.

## Materials and methods

### Study population

This study included clinical and imaging data of patients diagnosed with CSVD based on MRI and clinical evaluation between April and December 2022. Inclusion criteria comprised the following: (a) participants aged above four decades; (b) MRI evidence of typical white matter hyperintensities (WMH), with or without other characteristic CSVD imaging markers; (c) history of at least one vascular risk factor. The following conditions led to participant exclusion: (a) significant narrowing or complete blockage of major cerebral or neck arteries, verified through vascular imaging techniques; (b) demyelination from other causes, such as infections, genetic conditions, or metabolic disorders; (c) severe cardiac, renal, or pulmonary dysfunction; (d) other intracranial lesions, including tumors, trauma, vascular malformations, or hemorrhage; (e) poor image quality.

### MRI protocol and parameters

All patients underwent MRI scanning using a 1.5 T Siemens MAGNETOM Aera scanner. The neuroimaging protocol included three-dimensional T1-weighted imaging (T1WI), T2-weighted imaging (T2WI), T2 fluid-attenuated inversion recovery (FLAIR), and time-of-flight magnetic resonance angiography sequences. The 3D T1WI was acquired using the following technical specifications: repetition time (TR) = 2000 ms, echo time (TE) = 2.84 ms, slice thickness (SLT) = 1 mm, matrix = 256 × 256, field of view (FOV) = 24 × 24 cm^2^. For the T2 FLAIR sequence, the acquisition parameters included: TR = 6,500 ms, TE = 95 ms, SLT = 5 mm with 1 mm inter-slice spacing, FOV = 24 × 24 cm^2^, matrix = 256 × 256.

### Image analysis

#### WMH Fazekas scoring

**Figure 1:**
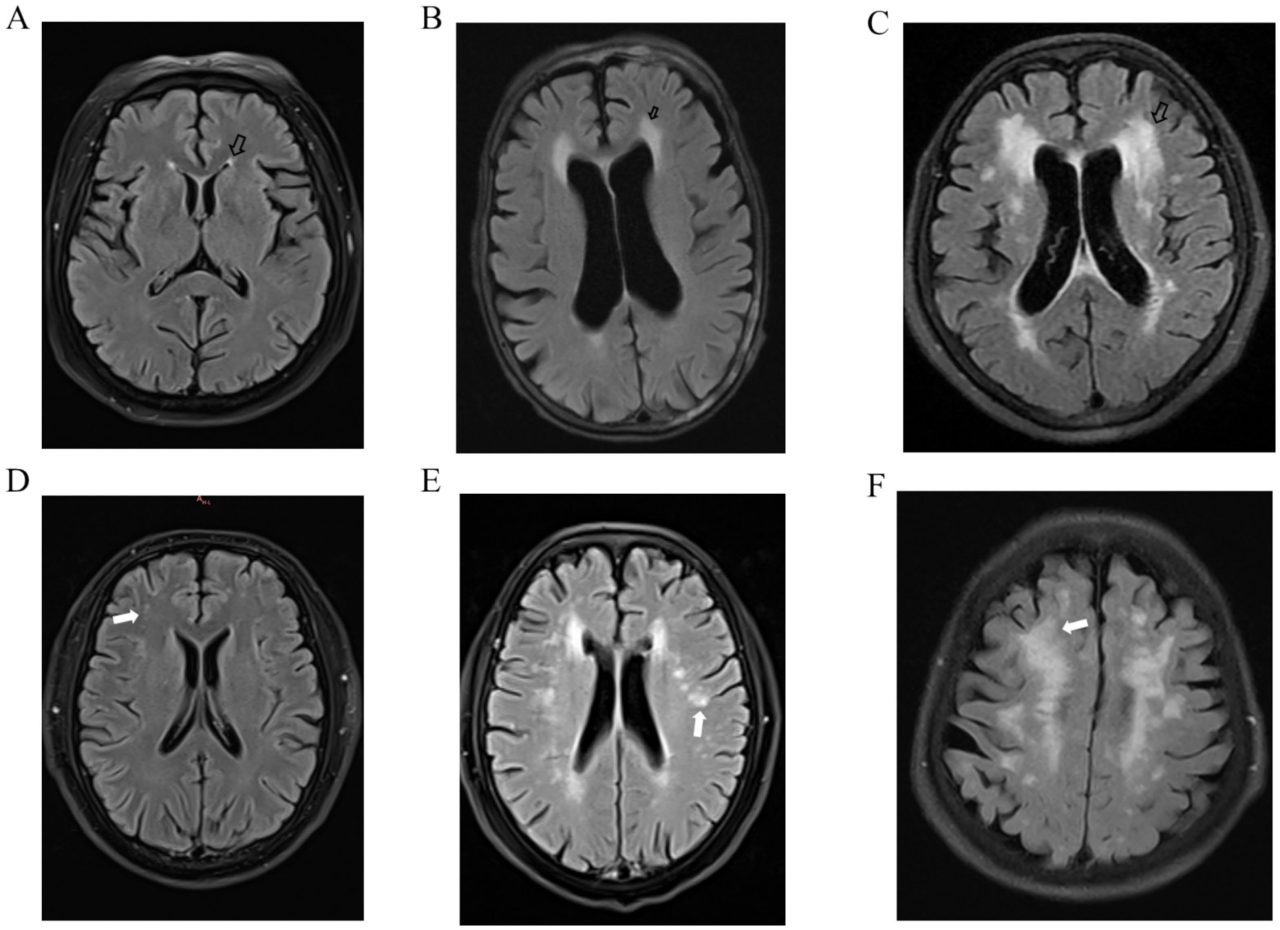
Representative neuroimaging illustrations demonstrating the Fazekas grading system for white matter hyperintensities. Black arrows indicate periventricular white matter hyperintensity Fazekas score of 1 **(A)**, 2 **(B)**, and 3 **(C)**; white arrows indicate deep white matter hyperintensity Fazekas score of 1 **(D)**, 2 **(E)**, and 3 **(F)**.

WMH were defined as regions demonstrating increased signal intensity on T2 FLAIR images, localized in either periventricular or deep white matter. The severity of both DWMH and PWMH was quantified according to the standardized Fazekas scoring system. This grading scheme employs a four-point ordinal scale ranging from 0 to 3, where score 0 indicates absence of detectable WMH, and score 3 corresponds to severe, confluent WMH (see Figure 1). Scoring was performed by reviewing whole-brain axial FLAIR images across all slices.

Two board-certified neuroradiologists (FW and ZX, each with >5 years of neuroimaging experience) independently performed Fazekas scoring while blinded to clinical and volumetric data. Inter-rater reliability was excellent, with weighted *κ* values of 0.931 for PWMH and 0.907 for DWMH. Any discrepancies were adjudicated by a senior neuroradiologist (XL, >15 years’ experience) through consensus review.

#### Normalized gray matter volume

The GM_n was employed as a volumetric measure, representing the proportional volume of gray matter volume relative to total intracranial capacity. First, the 3D T1WI images were converted from DICOM to NIfTI format. The fsl_anat pipeline within the FSL software was used to automatically generate gray matter and intracranial labels across the entire brain volume. Finally, these whole-brain, tissue-specific labels were then utilized for volumetric computations, with GM_n derived through division of gray matter volume by total intracranial volume (21).

### Statistical analyses

Descriptive statistics for normally distributed continuous variables were expressed as mean ± standard deviation, while non-normally distributed variables were presented as median (interquartile range, IQR). Categorical data were reported as frequencies. Differences in GM_n across different Fazekas scores for DWMH (PWMH) were analyzed using the Kruskal–Wallis test. To reduce the risk of type I error due to multiple comparisons, post-hoc pairwise group comparisons were performed using Wilcoxon rank-sum tests, and *p*-values were adjusted using the false discovery rate (FDR) correction. To account for potential confounding effects, a multivariate regression model incorporating established vascular risk factors was conducted to identify factors independently associated with GM_n. To further explore the potential mechanistic pathway linking age and gray matter atrophy, a mediation analysis was conducted using the PROCESS for Statistical Package for Social Sciences (SPSS) (Model 4). We tested whether PWMH (Fazekas score) mediates the effect of age on GM_n, with adjustment for sex, vascular risk factors, and DWMH score. The significance of the indirect (mediated) effect was evaluated using 5,000 bootstrapped samples. Statistical analyses were performed using SPSS version 20.0. A *p*-value < 0.05 was considered statistically significant.

### Results

A total of 125 patients with white matter hyperintensities were included in the study. Of these, 57 (45.6%) were male, with a mean age of 59 ± 11 years. The prevalence of vascular risk factors included hypertension in 67 (53.6%) patients, diabetes in 22 (17.6%), smoking in 27 (21.6%), and hyperlipidemia in 31 (24.8%). The median (IQR) of GM_n was 0.23 (0.22, 0.24). Baseline characteristics are summarized in Table 1.

**Table 1:** Baseline characteristics of the study population.

Variable	*n* = 125
Male, *n* (%)	57 (45.6)
Age, years, mean ± SD	59 ± 11
Hypertension, *n* (%)	67 (53.6)
Diabetes, *n* (%)	22 (17.6)
Smoking, *n* (%)	27 (21.6)
Hyperlipidemia, *n* (%)	31 (24.8)
PWMH, *n* (%)	
1	86 (68.8)
2	31 (24.8)
3	8 (6.4)
DWMH, *n* (%)	
0	24 (19.2)
1	74 (59.2)
2	18 (14.4)
3	9 (7.2)
GM_n, median (IQR)	0.23 (0.22, 0.24)

### Comparison of GM_n across different DWMH Fazekas scores

**Figure 2:**
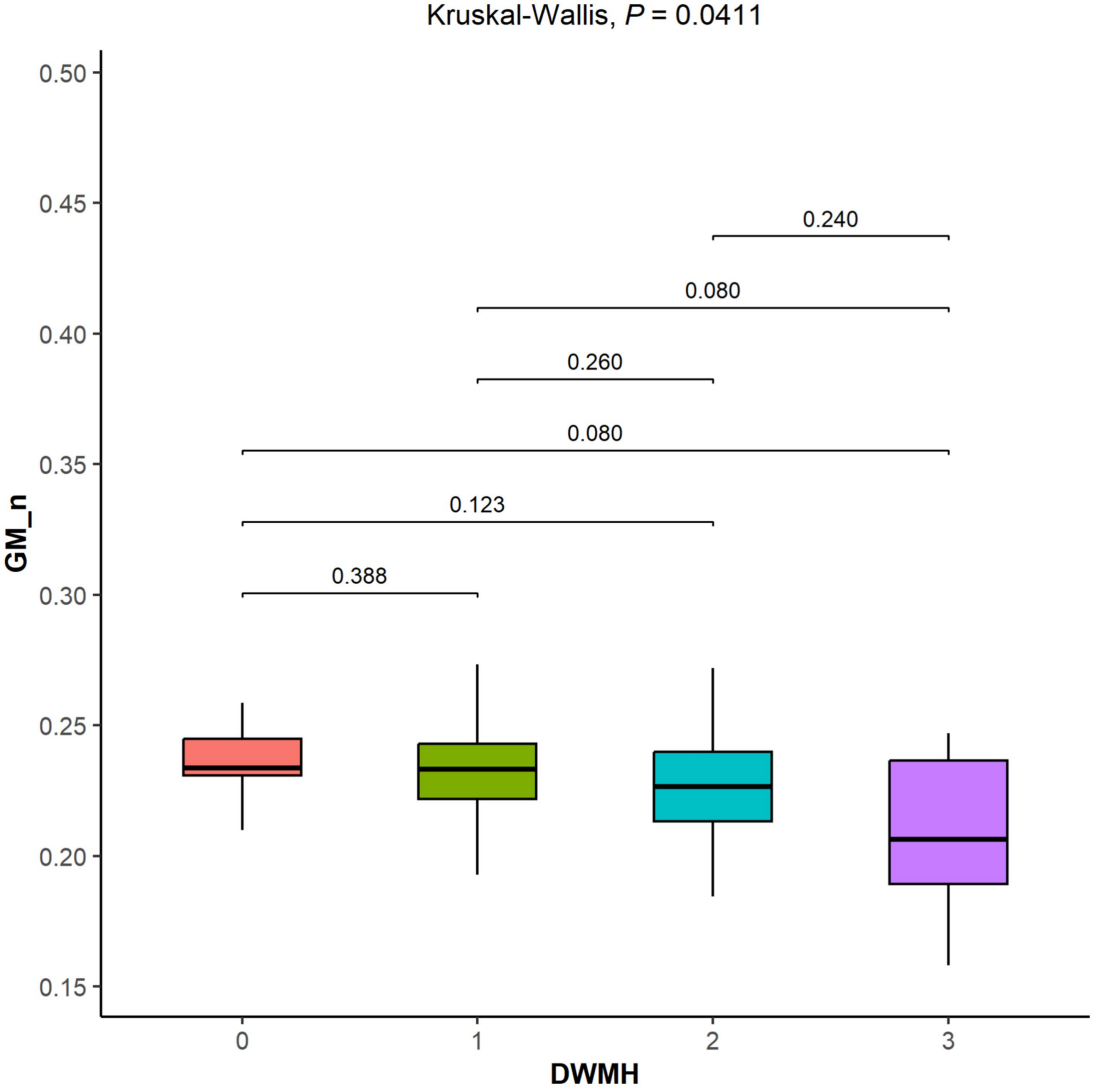
Comparative analysis of Fazekas scores for deep white matter hyperintensity (DWMH) and normalized gray matter volume (GM_n). Groupwise comparisons were assessed using Wilcoxon tests with false discovery rate (FDR) correction. Kruskal–Wallis testing showed a marginally significant group-level difference (*p* = 0.041). However, no pairwise comparisons remained statistically significant after FDR correction. A trend toward lower GM_n was noted for DWMH score 3 compared to those with scores 0 and 1.

Kruskal–Wallis testing revealed a marginally significant group-level difference in GM_n across DWMH Fazekas scores (*p* = 0.041). However, subsequent *post hoc* pairwise comparisons did not reach statistical significance after FDR correction. A trend toward lower GM_n was observed in patients with DWMH score 3 compared to those with scores 0 and 1 (FDR-adjusted *p* = 0.08), but this was not statistically significant (see Figure 2).

### Comparison of GM_n across different PWMH Fazekas scores

**Figure 3:**
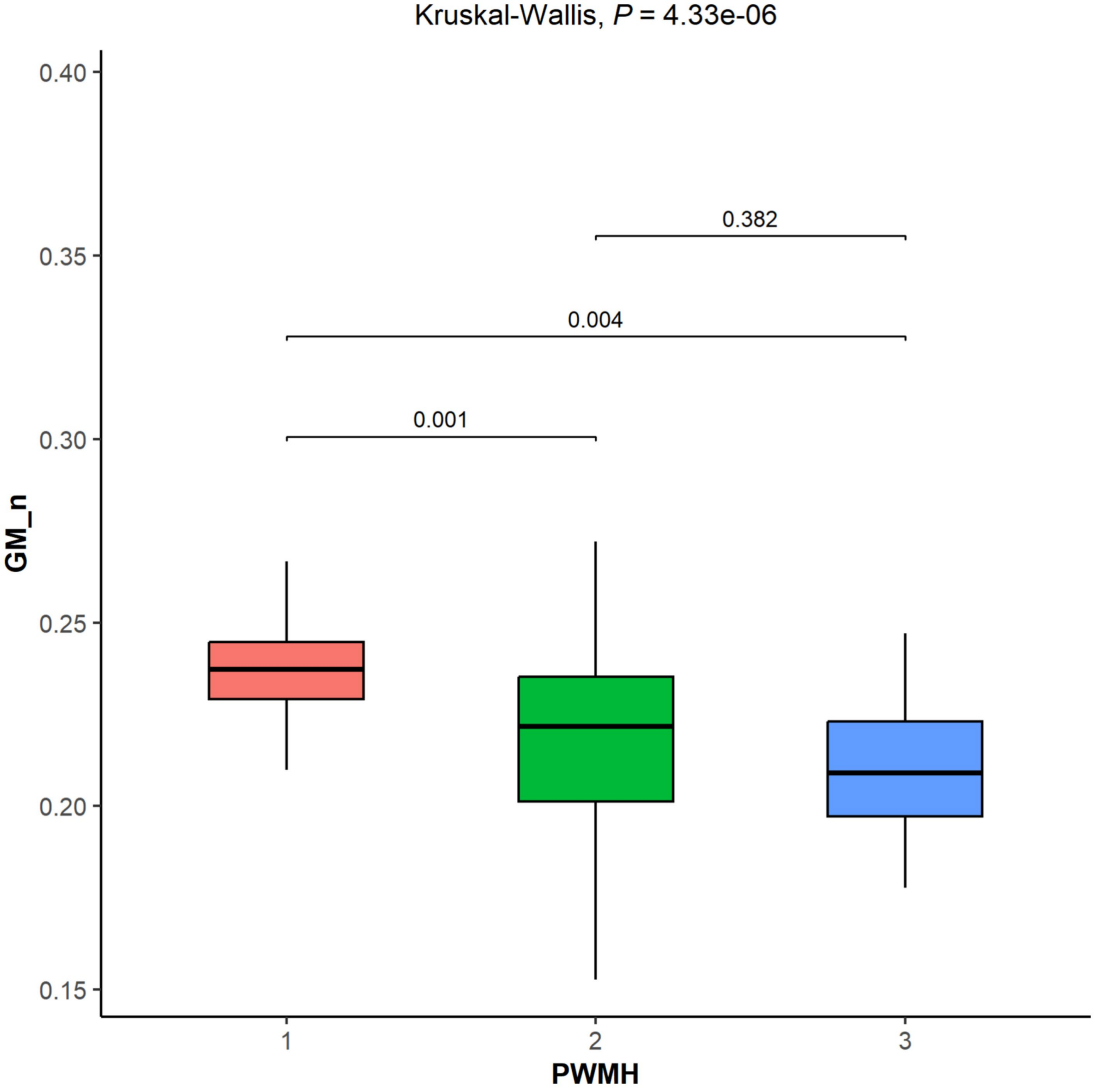
Comparative analysis of Fazekas scores for periventricular white matter hyperintensity (PWMH) and normalized gray matter volume (GM_n). Groupwise comparisons were assessed using Wilcoxon tests with FDR correction. Kruskal–Wallis testing revealed a significant overall difference (*p* < 0.001). FDR-adjusted pairwise comparisons showed that both PWMH score 2 and 3 groups had significantly lower GM_n compared to score 1 (*p* = 0.001 and 0.004, respectively). No significant difference was observed between score 2 and score 3 (*p* > 0.05).

Statistical analysis revealed a significant difference in GM_n across different Fazekas scores for PWMH (*p* < 0.001). Pairwise comparisons showed significant differences in GM_n between the PWMH Fazekas score 2 and 3 groups and the score 1 group (FDR-adjusted *p* = 0.001, 0.004, respectively). However, no significant volumetric difference was found between the Fazekas score 2 and 3 groups (*p* > 0.05) (see Figure 3).

### Multivariate analysis of factors affecting GM_n

After adjusting for vascular risk factors (hypertension, diabetes, smoking, hyperlipidemia), multivariate analysis indicated that age and PWMH Fazekas score were independently associated with GM_n (*p* < 0.05). The *β* coefficients (95% confidence intervals) for age and PWMH Fazekas score were −0.212 (−0.412, −0.011) and −0.408 (−0.605, −0.210), respectively, as shown in Table 2.

**Table 2:** Multivariate analysis of factors influencing normalized gray matter volume.

Variable	*β*	*p*-value	95.0% confidence interval	
			Lower limit	Upper limit
Age	−0.212	0.039	−0.412	−0.011
Gender	−0.142	0.118	−0.321	0.037
Hypertension	0.107	0.215	−0.063	0.277
Diabetes	−0.095	0.230	−0.251	0.061
Smoking	−0.127	0.171	−0.309	0.055
Hyperlipidemia	−0.024	0.766	−0.180	0.133
PWMH	−0.408	<0.001	−0.605	−0.210
DWMH	0.002	0.982	−0.197	0.201

### Mediation analysis: PWMH as an intermediate link between age and GM_n

**Figure 4:**
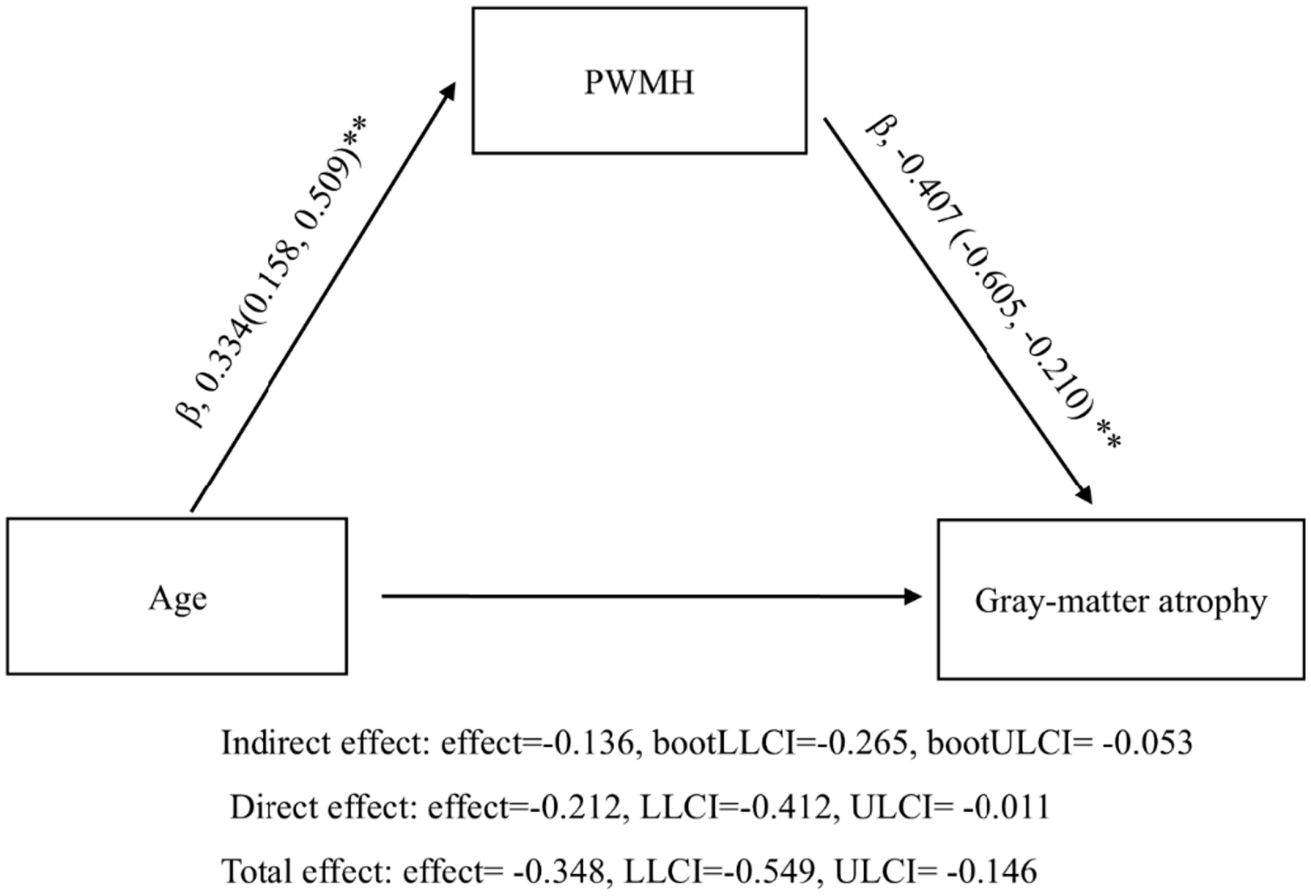
Mediation model illustrating the indirect effect of age on GM_n via PWMH (Fazekas score). The model was adjusted for sex, vascular risk factors, and DWMH (^**^p < 0.001).

To further investigate the pathway linking age to GM atrophy, we conducted a mediation analysis with PWMH severity as the mediator between age and GM_n. The results revealed a significant indirect effect of age on GM_n through PWMH. Specifically, path a (age → PWMH) was significant (*β* = 0.334, 95% CI: 0.158 to 0.509, *p* < 0.001), as was path b (PWMH → GM_n) (*β* = −0.407, 95% CI: −0.605 to −0.210, *p* < 0.001), controlling for gender, vascular risk factors and DWMH. The indirect effect was significant (*β* = −0.136, 95% CI: −0.265 to −0.053), accounting for 39.1% of the total effect (Figure 4).

## Discussion

GM atrophy is one of the hallmark features of neurodegenerative diseases and a major contributor to cognitive decline (13, 17). This study aimed to explore how different types of WMH, specifically PWMH and DWMH, affect GM_n. Our findings indicate that the severity of PWMH, not DWMH, is significantly associated with GM_n, and this association remains independent of vascular risk factors, such as hypertension, diabetes, and smoking. Although patients with DWMH score of 3 showed a trend toward reduced GM_n in univariate analysis, the association did not remain significant after adjusting for age and vascular risk factors. These results underscore the importance of distinguishing WMH subtypes when assessing structural brain changes in CSVD.

To further explore the mechanisms linking age and GM atrophy, we conducted a mediation analysis and found that PWMH significantly mediated the association between age and GM_n, independent of sex, vascular risk factors, and DWMH. This suggests that PWMHs are not merely correlates of brain aging but may represent a key intermediate step in the pathophysiological cascade from vascular aging to GM degeneration. Prior studies have reported similar mediation effects using total WMH burden (18). Our study extends these findings by demonstrating that PWMH specifically, rather than overall WMH burden, mediates this relationship. By focusing on WMH subtypes, we provide a more anatomically and mechanistically refined understanding of age-related GM atrophy in CSVD.

The distinct associations between PWMH and DWMH with GM_n may be explained by differences in their anatomical locations, fiber connectivity, and microvascular environments. PWMHs are typically located adjacent to the lateral ventricles and involve long-range association and projection fibers, such as the superior longitudinal fasciculus. Disruption of these tracts may impair interregional communication and trophic support, leading to transneuronal degeneration and subsequent GM atrophy (10, 22). WMH is known to damage axons and myelin sheaths, compromising structural connectivity and depriving neurons of metabolic and synaptic support (12). In contrast, DWMHs predominantly affect short U-fibers and local networks, which may explain their weaker association with global GM atrophy (22). Clinically, PWMH have been more consistently associated with cognitive impairment than DWMH (19, 20). Recent large-scale and longitudinal studies have further supported our findings. For example, Barisano et al. (23), using a population-based cohort of over 2000 elderly participants, demonstrated that PWMH burden—but not DWMH—was independently associated with dementia risk. Similarly, Liu et al. (24), in a longitudinal analysis, found that PWMH mediated the progression from subjective cognitive decline to dementia, while DWMH showed no such effect. These parallel findings across structural and clinical domains reinforce the notion that PWMH may play a more prominent role in associated with GM atrophy.

Microvascular factors may also contribute to the differential impact of PWMH and DWMH. Studies using dynamic contrast-enhanced MRI have demonstrated that PWMH regions exhibit higher blood-brain barrier (BBB) permeability (elevated K_trans_) and greater free water content compared to DWMH and normal-appearing white matter (16, 25). These features reflect more severe microstructural disruption, axonal degeneration, and impaired interstitial fluid clearance. Such changes may compromise synaptic plasticity and GM metabolic support, leading to progressive GM loss.

Our findings highlight the independent association between PWMH burden and GM atrophy, underscoring the importance of anatomical WMH subtypes in CSVD. Recent spatial and network-based studies provide additional perspectives to refine this classification. For instance, Beyer et al. (26) employed bullseye segmentation identified that anterior periventricular WMHs—an area overlapping with typical PWMH—were associated with elevated blood pressure, and cognitive decline. In contrast, juxtacortical-deep occipital WMHs—more representative of DWMH—were associated with incident all-cause dementia but not vascular risk factors. Although these findings did not directly assess GM atrophy, they support the notion of spatial heterogeneity in WMH pathophysiology. Furthermore, lesion network mapping (LNM) studies have shown that WMH connectivity to attention and executive control networks better predicts cognitive impairment than total WMH volume alone (27). Given their disruption of long-range projection fibers and central positioning within structural brain networks, PWMHs may exert a disproportionately greater impact on network integrity and subsequent GM atrophy compared to DWMHs. Future investigations integrating refined spatial classification (e.g., bullseye phenotypes), connectivity-informed metrics (e.g., LNM), and cognitive assessments are warranted to better delineate how WMH subtypes contribute to both gray matter loss and functional impairment in CSVD.

These spatial and connectomic findings converge with the broader perspective that CSVD is a whole-brain disease in which focal WM lesions can influence distant GM regions. For example, Wang et al. (28) reported that WMH-related GM atrophy shows region-specific patterns and is differentially associated with cognitive domains. Although that study did not differentiate PWMH and DWMH, their findings support the notion that white matter damage in strategic locations may have disproportionate effects on GM structure and function. Our study builds on this framework by highlighting that PWMH exerts broader and stronger effects on GM integrity, while DWMH may have more limited GM consequences. The observation that PWMH mediates age-related gray matter loss also provides a plausible mechanism for the widely reported link between PWMH and cognitive decline.

Several limitations should be acknowledged in this investigation. First, the relatively small sample size and single-center design may limit the generalizability of the findings. Second, the lack of region-specific analyses of GM, cognitive assessments, and diffusion imaging data, which limits our ability to clarify the anatomical, functional, and connectivity-based mechanisms by which PWMH and DWMH may contribute to GM atrophy. Third, the cross-sectional nature of our study design also limited our ability to assess temporal dynamics in the progression of WMH, axonal pathway degeneration, and gray matter volumetric changes. Subsequent investigations incorporating multicenter designs with larger sample sizes, advanced diffusion imaging protocols, cognitive assessments and longitudinal follow-up assessments are warranted to elucidate the temporal progression and pathophysiological mechanisms underlying these structural alterations.

In conclusion, this study demonstrates that PWMH, but not DWMH, is independently associated with GM atrophy in CSVD and mediates the effect of age on GM_n. The distinct anatomical connectivity, vascular vulnerability, and microstructural disruption of PWMH may underlie its greater contribution to GM degeneration. When assessing and managing CSVD patients, particular attention should be given to the severity of PWMH, especially in older individuals. Given the independent association between PWMH and decreased gray matter volume, early detection and intervention of PWMH may have significant clinical implications in preserving brain health and cognitive function.

## Data Availability

The original contributions presented in the study are included in the article/supplementary material, further inquiries can be directed to the corresponding author.
